# Re-thinking the evolution of microblade technology in East Asia: Techno-functional understanding of the lithic assemblage from Shizitan 29 (Shanxi, China)

**DOI:** 10.1371/journal.pone.0212643

**Published:** 2019-02-25

**Authors:** Yanhua Song, Stefano Grimaldi, Fabio Santaniello, David J. Cohen, Jinming Shi, Ofer Bar-Yosef

**Affiliations:** 1 Department of Archaeology, Shanxi University, Taiyuan, China; 2 Dipartimento di Lettere e Filosofia, Università degli Studi di Trento, Trento, Italy; 3 Istituto Italiano di Paleontologia Umana, Anagni, Italy; 4 Progetto Did@ct, Trento, Italy; 5 Department of Anthropology, National Taiwan University, Taipei, Taiwan; 6 Shanxi Museum, Taiyuan, China; 7 Department of Anthropology, Harvard University, Cambridge, Massachusetts, United States of America; Max Planck Institute for the Science of Human History, GERMANY

## Abstract

The lithic assemblage from Shizitan 29, a late Upper Paleolithic open-air site in Shanxi, China, provides evidence for the earliest, well-dated microblade production in East Asia, ca. 26/24 Ka cal BP. To pursue a behavioral rather than traditional typological understanding of this key adaptive technology, we apply a techno-functional approach that enables us to reconstruct the entire operational sequence in behavioral terms through the derivation of technical objectives. This methodology can serve as a model to be applied to other assemblages for greater understanding of the origins and spread of the broadly distributed eastern Asian Late Pleistocene microblade industries. Within the eight cultural layers at Shizitan 29, microblade production abruptly appears at the top of Layer 7 following earlier core-and-flake production, supporting hypotheses of microblade technology arising within adaptive strategies to worsening Late Glacial Maximum environments. Significantly, reconstruction of the operational sequence supports microblade technology being introduced into the North China Loess Plateau from regions further north. It also allows us to re-think microblades’ relationship in behavioral terms with earlier limited examples of East Asian blade production and the evolution and spread of microblade technology, providing new insights into the adaptive relationships between subsequent microblade productions.

## Introduction

While the Upper Paleolithic was originally defined, based on the European and southwestern Asian archaeological record, by the production of blades, blade production in early Upper Paleolithic eastern Asia is limited. A form of blade production, called microblade technology, does, however, become prevalent within a well-defined region of northern East Asia north of the Yellow River during the later half of the Upper Paleolithic. Understanding of the unique scenario of the emergence and spread of microblade technology can offer new insights into the nature of human behavioral evolution during the Late Pleistocene, and particularly how modern humans were equipped to adapt in northern latitudes to the worsening glacial climate and subsequent amelioration. This study addresses a specific issue concerning microblades as a human adaptation in Late Glacial Maximum (LGM) eastern Asia, suggesting a more complex scenario for the development of the final Upper Paleolithic in this broad region. As we discuss below, the reliance of the predominant analytical approach in East Asia—morphologically-based typology—primarily on the shapes of microcores limits ability to evaluate behavioral choices and processes in producing microblades. Here, using the exceptional dataset from the Shizitan 29 open-air site in Shanxi Province, China, we apply a techno-functional approach that enables us to reconstruct the entire operational sequence in behavioral terms. This approach, with its focus on the technical objectives of the tool maker rather than on final core shape typologies, offers new insights into the early stages of microblade production.

Shizitan 29 provides a diachronic record across the major change in Upper Paleolithic (also commonly termed the “Late Paleolithic” in the Chinese literature) lithic production from the core-and-flake assemblages of the lowermost cultural layers, Layer 8 and the base of Layer 7, to the appearance, ca. 26 Ka cal BP, and initial development of the earliest, well-dated microblade assemblage in China from the upper spits of Layer 7 and upward [1].

Shizitan 29 is not the only site representing this change, but other sites in North China of possibly similar date, such as Longwangchan [2–3], remain insufficiently reported, particularly from a technological perspective, and lack reliable dating. The large-area excavations and deeply stratified, systematically dated layers at Shizitan 29 also provide unique opportunities to investigate diachronic change in behavioral patterns related to lithic production, which we do in a combined approach featuring raw material exploitation and reduction sequence reconstructions.

Blade production belongs to a suite of technological and symbolic developments best understood from sites in Europe and southwestern Asia that mark the Upper Paleolithic and behavioral modernity. For lithics, blade production is taken as a key technological signifier of these changes that began ca. 45 Ka cal BP as part of an “Upper Paleolithic behavioral revolution” [4–5]. In other regions of Eurasia, because of the limited presence of blades and other “typical”—from a European perspective—indicators of behavioral modernity, the application of European-derived standards, as well as local analytical frameworks based primarily in lithic morphological typology, greatly impact interpretation of the onset and evolution of modern behavior. In China, for instance, while there are limited examples of blade production, the early Upper Paleolithic (ca. 40/35–25/26 Ka cal BP) primarily features core and flake industries; these can be defined as “Upper Paleolithic” technologically and behaviorally because they co-occur with varying amounts of polished bone tools, grinding stones and handstones likely for processing wild plants and perhaps ochre [1], and small amounts of body ornamentations (e.g., shell and eggshell beads) [1,6–8]: thus in sum the lithics can be seen to be part of distinctive material patterns of modern behavior in East Asia. Beginning ca. 26 Ka cal BP, however, northern China, which was originally occupied by makers of core and flake industries, becomes inhabited by the bearers of bladelet technology who produce what is known as “microblade industries” [1–2,9–12]. These microblade industries later spread into Korea and the Japanese archipelago [13–14].

Chronologically, microblades are not part of the earliest stages of Upper Paleolithic developments, and they usually are considered as a development of blade production, but this assumption has not been validated archaeologically and remains under-theorized in East Asia. With blade production never dominating across the eastern Eurasian landscape before the appearance of microblades during the LGM, such as at Shizitan 29, however, we must also try to understand microblade technology as deriving out of local cultural trajectories that were different from those of western Eurasia [15–16]. This understanding of the evolution of this technology requires parallel techno-functional analyses to that which we are introducing to the region here through this study of the Shizitan 29 assemblage, with the intent that this approach can then be performed on other assemblages: these together can provide the deeper insights into technical objectives that are needed to state if there is or is not a techno-functional relationship between blades and bladelets/microblades. This study introduces the techno-functional approach, and because we are looking at the earliest microblade production in a region with little or no prior blade production (depending on how broadly we look), in technological and functional terms there is no reason to first make an arbitrary dimensional distinction that forces us to assume that there are techno-functional distinctions between blade and microblade production. Although this runs counter to typologically-based approaches, the consideration of technical objectives across the entire reduction sequence in the techno-functional approach requires us to first lift away this arbitrary width-measurement based distinction between the two. Microblade production continues through the Terminal Pleistocene, when it becomes a key component of numerous Upper Paleolithic sites across a wide geographical distribution from North China to Siberia, Mongolia, the Russian Far East, Korean Peninsula, and Japanese Archipelago, as well as in North America, particularly during the Younger Dryas [17–20].

### Shizitan 29

**Fig 1:**
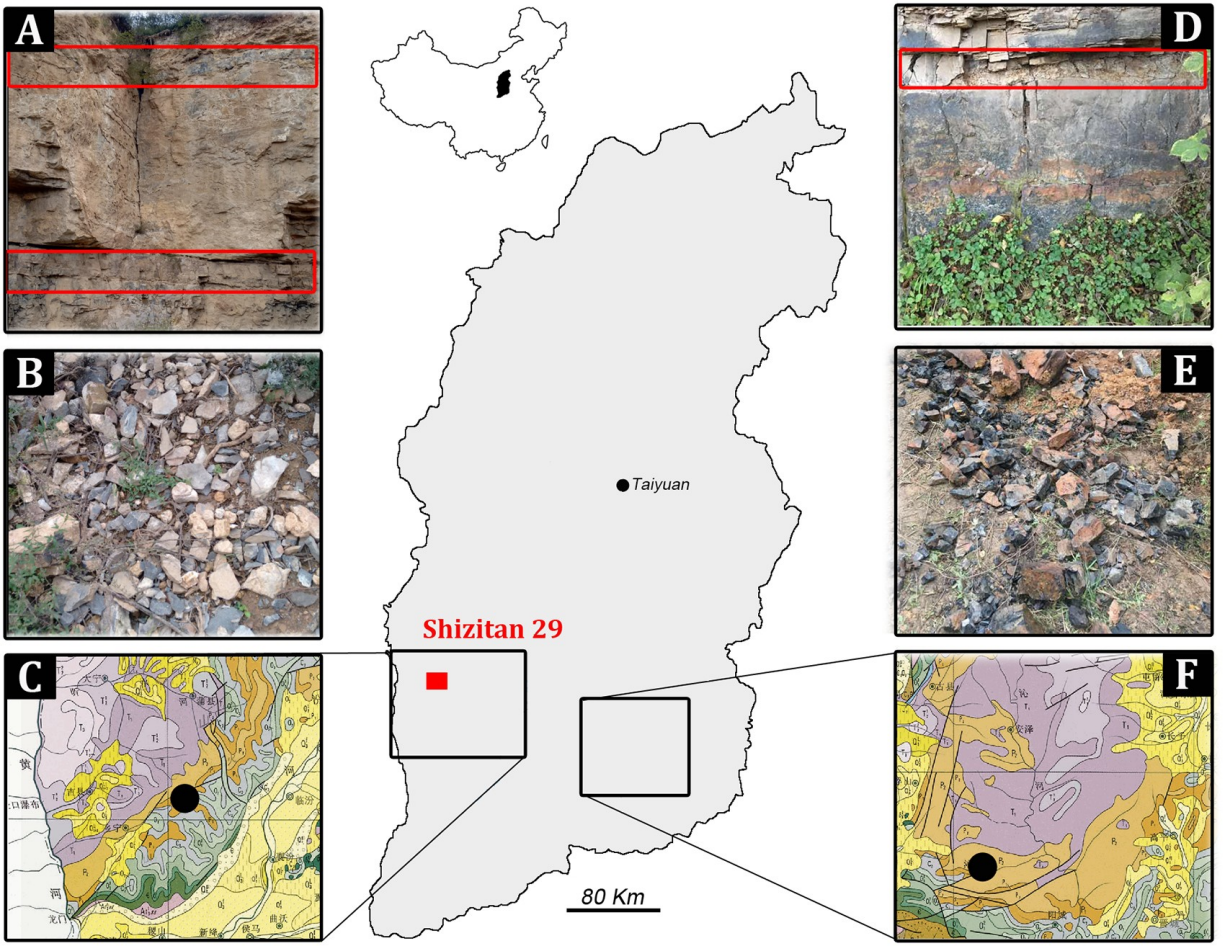
The location of the Shizitan 29 site and its geological setting. (red rectangle) Location of the Shizitan 29 site in Shanxi province and (black rectangles) the locations of two areas of the province where Permian flint deposits are distributed from which Black flint was sampled (black circles in C and F). Black flint (A and D) in primary deposition and (B and E) naturally eroded.

The Shizitan 29 site (36° 2′ 54″ N, 110°35′ 22″ E, 723 m above sea level) is an open-air site located approximately 500 m east of Shizihe Village, Shanxi Province (Fig 1). Excavation opened an area of 1,200 m^2^, exposing a depositional sequence 15 m deep that included eight cultural layers, with Level 8 being the lowest. Findings, including 285 excavated hearths (but none in Layer 8) and more than 80,000 artifacts, provide a unique, rich dataset [1, 21]. Micromorphology shows that Layer 8, predating the Last Glacial Maximum, is colluvial, more clayey overall, and extensively bioturbated, likely by grass rooting. Layer 7 is an aeolian deposit with weakly developed calcareous features that might point to somewhat dryer conditions. In the uppermost six cultural layers, especially Layer 6, artifacts and hearths were generally preserved within reworked loess deposits.

Layer 8, according to AMS ^14^C data, is dated around 28 Ka cal BP. Three dates from the top of Layer 7 range from ca. 24 to 26 Ka cal BP, during the Last Glacial Maximum, and these dating samples are associated with the earliest appearance of microblade technology. Pollen, non-pollen palynomorph (NPP), soil micromorphology, and faunal analyses show that Layer 8 features a warmer and damper climate than later LGM conditions, with soil formation, warm-loving, deciduous species, and one hunted deer species. Layer 7 pollen and NPP spectra show gradual climatic change at the beginning of LGM with increasing amounts of *Pinus* and *Artemisia annua*, while amongst faunal remains, *Equus caballus*/*E*. *hemionus* appear, indicating a steppe or encroaching semi-desert environment. Layers 6–4 (ca. 24–19.5 Ka cal BP) demonstrate further cooling and drying with dark coniferous species appearing and with *Artemisia annua* and Chenopodiaceae pollen predominant among the herbaceous species.

While this study focuses on a techno-functional approach to the appearance of microblades at Shizitan 29, this appearance must be considered within the overall environmental and cultural contexts of this major behavioral shift among hunter-gatherer groups and the nature of their activities at the site. In addition to blades, behavioral modernity at the site is also reflected in associated cultural features such as early examples of the processing of starchy plant foods [22], grasses, and pigments with grinding stones and handstones (appearing from Layer 8 and upward), ornaments of shell and ostrich eggshell [23–25], and polished bone needles that could be used to sew clothing [26]. The 285 hearths appear in clear linear arrangements likely related to the river channel and perhaps reflect new pyrotechnological practices and a new form of repeated, specialized usage of the site over a series of ephemeral occupations [1,22].

## Methodology

### The technological approach

The study of the Shizitan 29 lithic assemblage follows an established methodology applied to Paleolithic assemblages in Western Europe [27–31] that stresses behavioral considerations based in “technical objectives” with this being the first application of this methodology to North China lithic production. We aim not only to provide a schematic model of the reduction sequences of the analyzed stone assemblage but also to clarify the relations between raw material variability, tool morphologies, and core reduction processes. We stress that reconstruction of the reduction sequence must not be the ultimate goal of a technological analysis. Instead, the reduction sequence serves as a methodological tool to identify adaptive responses by the human group, typically to local environmental constraints. We reconstruct the reduction sequence to identify what we call the “technical objectives” pursued by prehistoric humans. Without consideration of technical objectives, any reconstructed reduction sequence remains empirical description useful only for better categorization of data through a simple terminology (i.e., Levallois flake, rejuvenation flake, blade, and so on).

Furthermore, in this paper, we employ the terminology “blade” [32] in reference to any elongated, morphologically standardized blank showing parallel or sub-parallel edges and scars. No further sub-classification based only on a width dimensional difference is made here. As a consequence, contrary to the arbitrary distinctions made in other studies of East Asian microblade production, which are focused on formal typology, we do not assume any distinction between blade and microblade based merely on form (width difference): such a distinction only should be made if a dimensional limit useful in distinguishing some technological or functional difference between these two groups is established, and this would be dependent on particular research questions. In the literature (an overview can be found in the Discussion section), a dimensional limit remains commonly in use but is subjectively defined and arbitrarily applied in the traditional typological approach without a clear explanation of its technological and functional meaning. In this paper, because our focus is on the *productive features* associated with the production of elongated blanks, we do not make any arbitrary dimensional distinction: this allows better evolutionary insight to the *initial production* of the small blades classifiable as microblades, i.e., we can gain better insight into how microblade production first began. Our final data support this approach, with our analysis of the Shizitan 29 lithics showing that a real metric or morphological distinction is absent, particularly for the questions we ask concerning the early appearance of microblades.

### The hypothetical reduction sequence

One goal of the technological approach is the identification of the technical objectives of the lithic production, and the identification of technical objectives requires consideration of each lithic from the assemblage within a hypothetical reduction sequence. We interpret any lithic as being a product of one stage of this sequence. It should be stressed that the attribution of each item to a particular stage should not be considered a mechanical procedure based on the recognition of its morphology. Instead, attribution mainly depends on: a) the analysis of the technical features shown by the whole assemblage; b) quantitative and metrical data characterizing the assemblage; c) the functional characteristics of the technical objectives; and d) experimentally-derived criteria. The hypothetical reduction sequence here is subdivided into four stages, as follows:

**(A) Early stage**. This is the beginning of the reduction sequence: raw material is imported into the site showing its natural morphology and dimensions. Items classified to this stage could include cores showing only one or two scars (such as from quality testing) or flakes with cortical or almost cortical surfaces that still reveal (sometimes partially) the original shape of the raw material. Here, some products showing clear traces of anvil percussion (such as half pebbles) also have been included in this stage.
**(B) Primary preparation**. This stage mainly involves blanks. Here, the role of the blanks is to shape a core that can ensure the production of the technical objectives; accordingly, products show “coarse” techno-morphological features evoking the next stage.
**(C) Full production**. This stage relates directly to the production of the technical objectives. If blades are the aim of the production, they would usually be identified not only by their standardized morphology and parallel edges, but also by parallel nervures. Opposed to this, if flakes are the aim of the production, “predetermined” blanks [30] would be identified. Cortex distribution is limited or totally absent. This stage usually ends with the total exhaustion or abandonment of the core. In some cases, re-preparation of the core may be needed during this stage, in which case the dimensions of the core are reduced. Due to the technically specialized morphology that the core must acquire in this stage, such re-preparation aims to maintain the original core shape. Then, rejuvenating blanks related to this stage (such as *tablettes*, *outrepassée* blades, or *débordant* flakes) should bear residual scars characterizing the Stage C products.
**(D) Final production**. At this stage, the previous production is no longer possible because the core’s features (size, morphology, etc.) no longer allow predetermined products similar to those from Stage C to be obtained. However, if the core is not completely exhausted, its exploitation may continue, but now, a change in both the technical objectives and the morphology of the cores occurs. Here, blanks will present technical features (e.g., orientation of the scars, thickness, irregular edges, etc.) that drastically modify the original full production stage morphology. Another option may occur when anvil percussion technique is applied or if the production aims to obtain flakes: in such cases, Stage D may follow directly from Stage A or Stage B.

### Lithic and raw material sample

A total of 74,735 stone artifacts were recovered at Shizitan 29, including 166 pieces from Layer 8 and 42,928 from Layer 7. This study (carried out September 2016 and October 2017 in Taiyuan, Shanxi) analyzed all lithics from Layer 8. From Layer 7, we analyzed all the cores and retouched flakes from the entire excavated area, but because of the large quantities of other items (due to the thick cultural accumulations across 1,200 m^2^ for this layer), we selected a sampling zone of 150 m^2^ (from the one meter units ranging 60–70 North and 85–100 East on the excavation grid) from which we analyzed all lithics. This sampled area represents the part of the site where the thickest anthropogenic deposits are found, is where radiocarbon and pollen samples were collected, and is present in the published profiles [1]. Excavation by arbitrary spits of 10 cm thickness within the cultural layers allowed vertical control for identifying the first appearance of blades in the top of Layer 7. For this reason, we divided Layer 7 into two sampling units for analysis, Layer 7 Top (spits 1–2, where the earliest microblade production appears) and Layer 7 Base (spits 7–12, where a core-and-flake production was found, as also in Layer 8, below it).

Raw material provenience was analyzed by comparison to geological sources. Although some suggest otherwise [33–34], outcrops of good quality flint are present in North China, with the southern part of Shanxi Province providing several types of workable flint as well as local sources of chert, limestone, quartzite, and quartz. Excavations at Shizitan 29 and other localities show that two types of good quality flint were exploited in prehistory, here called “Black flint” and “River flint”. Black flint is found in Permian deposits labeled as P1 and P2 on the standard Shanxi geological map [35]. Black flint outcrops—visited for this study—are located 30–40 km east of Shizitan (Fig 1a–1c) and are composed of thick layers of dark grey or black flint; abundant, small blocks can be easily collected close to outcrops as they may be found fallen or eroded from the geological layer. The flint quality is very good, but it is often affected by brittle deformations (fractures) due to tectonic stress; consequently, dimensions of suitable blocks are generally reduced. Other outcrops of Black flint are attested about 150 km from Shizitan, in the Permian formations in southeastern Shanxi (Fig 1d–1f). For River flint, rounded pebbles of several centimeters length are typically found along the Yellow River’s banks [1] beginning at 9.5 km from the site; broadly speaking, this translucent, multicolored lithotype has an overall good knapping attitude. Other relevant raw materials found along the riverbank are pebbles of quartz and quartzite. Chert, limestone, sandstone, and other stones are less relevant in the Shizitan archaeological record.

## Results

### Core-and-flake assemblage (Layer 8 and Layer 7 Base)

**Fig 2:**
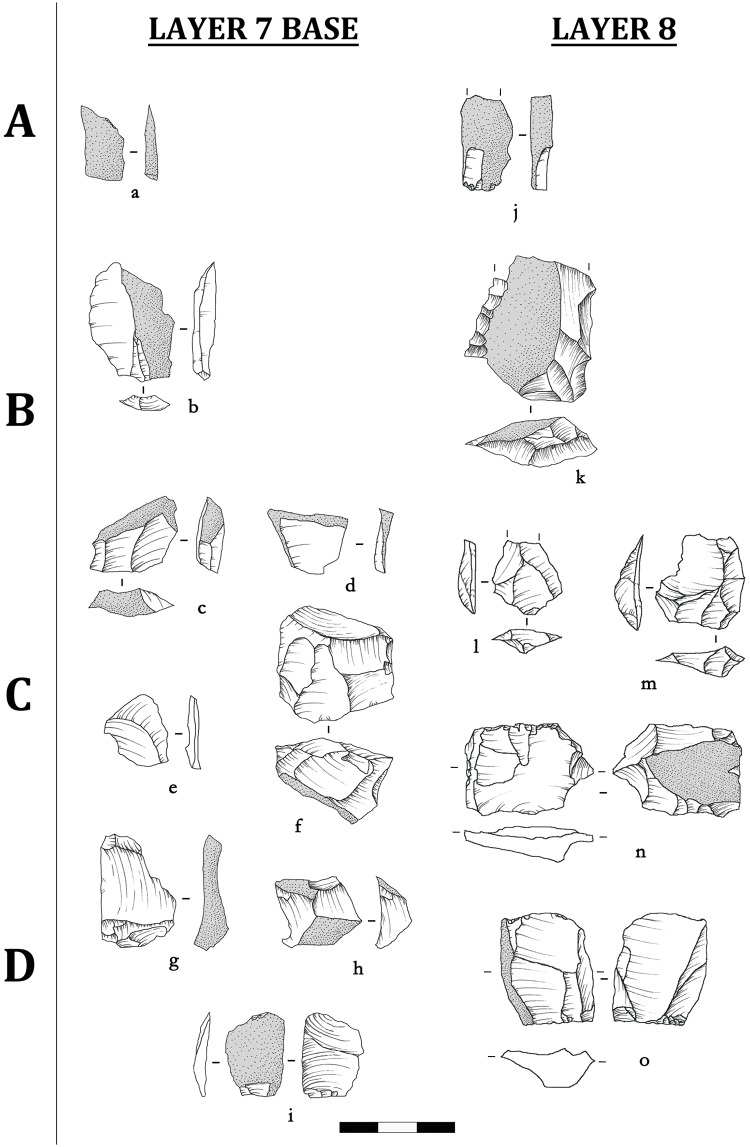
Shizitan 29: Reduction sequence of the Core-and-flake assemblage. Lithic drawings showing reduction stages for Black flint lithic industry from (a-i) layer 7 Base and (j-o) layer 8.

**Fig 3:**
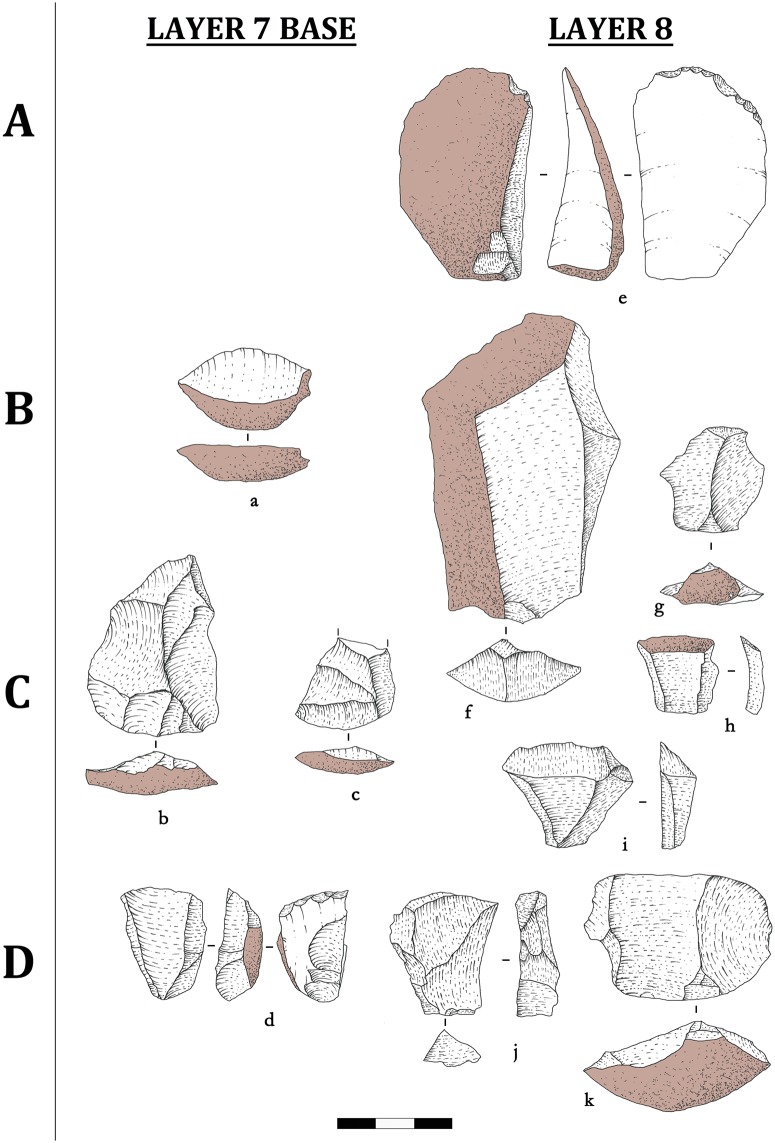
Shizitan 29: Reduction sequence of the Core-and-flake assemblage. Lithic drawings showing reduction stages for Quartzite lithic industry from (a-d) layer 7 Base and (e-k) layer 8.

A core-and-flake lithic assemblage is found in Shizitan 29’s earliest Layer 8 and continues into Layer 7 Base. Black flint (Fig 2) and quartzite (Fig 3) are the most exploited raw materials, while River flint appears only in very low frequencies (Tables 1 and 2). The Black flint core to flake ratio ranges from 4 to 6 flakes per core, while quartzite cores show higher values, from 10 to 15 flakes per core. It is also worth mentioning that, broadly speaking, cores do not seem to be hyper-exploited regardless of raw material. Evidence of anvil technique is largely observed in these two core-and-flake assemblages, reaching 23% among cores and flakes. This could explain the larger amount of flakes per core made from quartzite. As evidence for anvil technique is naturally underestimated in lithic analysis, it is possible to assume that this technique was largely adopted, although it still remains difficult to establish the reasons for this.

**Table 1:** Shizitan 29, layer 8 and 7: The lithic sample.

Layers	Raw material	Cores	Blades	Flakes	Other	Total	Sampling regime
**7 Top**	**BF**	33	106	77	16	**232**	*All cores and all retouched flakes*. *All blades*, *and all recorded flakes from the selected area*
	**RF**	27	112	57	7	**203**	
	**Q**	7	2	39	5	**53**	
	**O**	0	10	2	4	**16**	
		**67**	**230**	**175**	**32**	**504**	
**7 Base**	**BF**	9	0	43	6	**58**	*Entire collection from the selected area*
	**RF**	1	1	10	3	**15**	
	**Q**	3	0	45	6	**54**	
	**O**	0	0	3	1	**4**	
		**13**	**1**	**101**	**16**	**131**	
**8**	**BF**	9	0	60	13	**82**	*Entire collection*
	**RF**	2	0	5	1	**8**	
	**Q**	3	0	32	5	**40**	
	**O**	0	0	1	0	**1**	
		**14**	**0**	**98**	**19**	**131**	
**Total**		**142**	**287**	**473**	**82**	**984**	

Quantitative data for the lithics from the three analyzed stratigraphic units from Shizitan 29, Layer 8, and 7 (Base and Top), and their sampling regimes: (BF) Black flint; (RF) River flint; (Q) quartz/quartzite; (O) other materials). The blade fragment found within layer 7 Base probably resulted from a limited post-depositional process repositioning it.

**Table 2:** Shizitan 29, layer 8 and 7 (base): The retouched lithic sample.

		Composites	Denticulates	Endscrapers	Esquillés	Marginal retouch	Notches	Points	Scrapers	
**7 Base**	**BF**	0	0	0	2	1	0	0	2	5
	**RF**	0	0	0	1	1	0	0	0	2
	**Q**	0	0	1	1	3	3	1	5	14
	**O**	0	0	0	0	0	0	0	1	1
		0	0	1	4	5	3	1	8	**22**
**8**	**BF**	0	0	0	3	2	0	0	4	9
	**RF**	0	0	0	1	0	1	0	0	2
	**Q**	1	2	0	0	0	0	0	1	4
	**O**	0	0	0	0	0	0	0	0	0
		1	2	0	4	2	1	0	5	**15**

Typological frequencies of retouched flakes from Shizitan 29, layers 8 and 7 Base according to raw materials: (BF) Black flint; (RF) River flint; (Q) quartz/quartzite; (O) other materials.

Flake butts are mostly flat (about 40%), but great variability in butt types is found. Broadly speaking, this could suggest high technical variability during the production of flakes, such as from knapping mistakes or re-preparation of the cores. Cortical or natural butts are not found on Black flint flakes: this could suggest that thick flakes might have been imported into the site to be used as cores. Thirty-seven flakes from Layers 8 and 7 Base are retouched; from a typological point of view, their distribution across eight categories of retouch shows a very limited variety of formal types. Scrapers, flakes with marginal retouch, and splintered (*esquillées*) flakes represent the most common types.

All 27 cores could be attributed to a reduction stage; for flakes, in Layer 8, we could attribute more than 60% of the flakes to a reduction stage, and for Layer 7 Base, 47% of the flakes (Table 3). Stage A is not represented among the cores from Layer 8, and Stages B and C are poorly represented; most of the cores (10 out of 14) belong to Stage D. This distribution seems to suggest very intensive exploitation activity. For the attributed flakes, just under 60% are in Stages C and D, while Stage A is represented by 24.7%; Stage B is poorly represented (15.6%). Black flint is the most abundant material for flakes in Stage A, and these are mainly cortical flakes.

**Table 3:** Shizitan 29, layer 8 and 7 (base): The reduction sequence.

**Cores**	**Stage A**		**Stage B**		**Stage C**		**Stage D**		
**Layer**	**8**	**7 Base**	**8**	**7 Base**	**8**	**7 Base**	**8**	**7 Base**	
**BF**	0	2	1	3	1	1	7	2	**17**
**RF**	0	0	0	0	0	0	0	1	**1**
**Q**	0	1	0	1	1	0	2	1	**6**
**O**	0	0	1	0	0	0	1	1	**3**
	**0**	**3**	**2**	**4**	**2**	**1**	**10**	**5**	**27**
**Flakes**	**Stage A**		**Stage B**		**Stage C**		**Stage D**		
**Layer**	**8**	**7 Base**	**8**	**7 Base**	**8**	**7 Base**	**8**	**7 Base**	
**BF**	9	4	6	3	12	8	7	5	**54**
**RF**	8	1	2	1	0	2	2	1	**17**
**Q**	1	4	0	5	10	11	4	2	**37**
**O**	0	0	0	0	0	1	0	0	**1**
	**18**	**9**	**8**	**9**	**22**	**22**	**13**	**8**	**109**

Attributed reduction stages of flakes and cores from Shizitan 29, layers 8 and 7 Base according to raw materials: (BF) Black flint; (RF) River flint; (Q) quartz/quartzite; (O) other materials.

The above attributions appear to indicate that the entire reduction sequence took place on site: Black flint seems to be imported into the site in its natural morphology (irregular nodules or parallelepiped blocks), although large and thick flakes may occasionally have been imported and knapped as a core. The techniques adopted for flake production are direct percussion combined with anvil percussion, and the flakes show a low level of morphological standardization. No clear evidence of functional specialization is observed. Furthermore, the low frequencies of retouched tools in association with the high frequency of unretouched flakes point toward lithic production in Layers 8 and 7 Base mainly oriented toward flakes used as general tools for different activities.

### Blade assemblage (Layer 7 Top)

**Fig 4:**
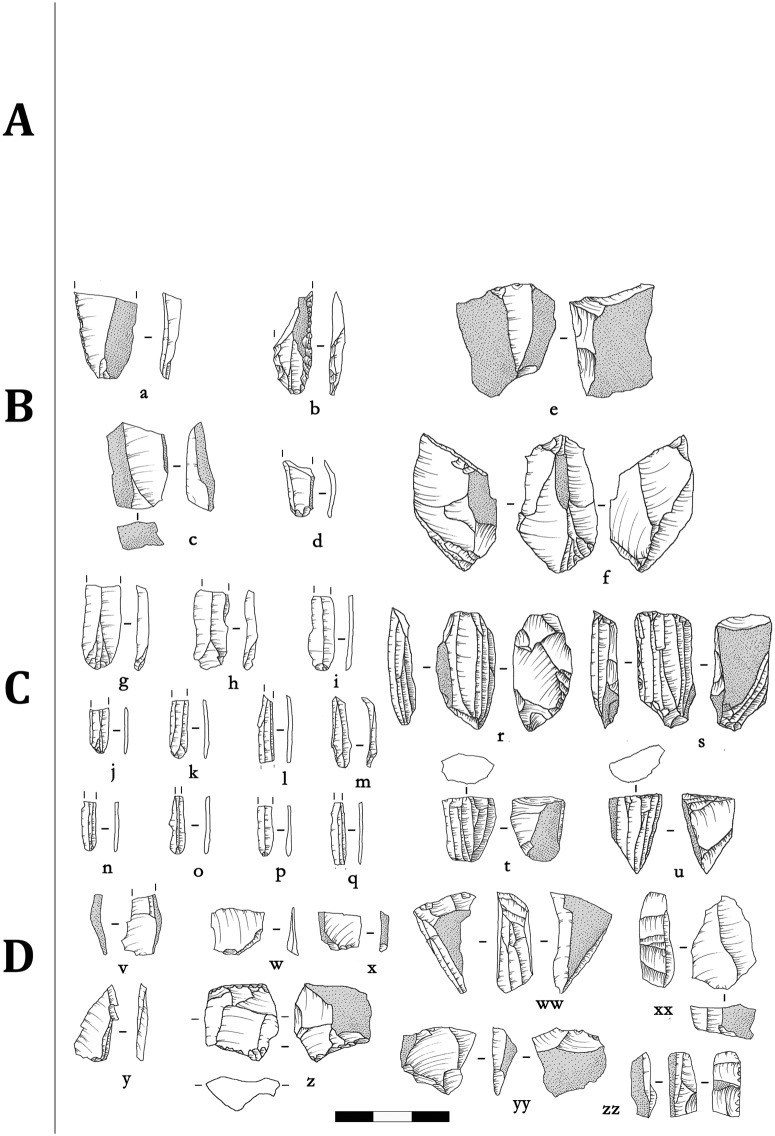
Shizitan 29, layer 7 Top: Reduction sequence. Lithic drawings showing reduction stages for Black flint lithic industry from layer 7 Top. (a-d) Flakes and (e-f) cores from Stage B; (g-q) Blades and (r-u) cores from Stage C; (v-y) Flakes and (z-zz) cores from Stage D.

**Fig 5:**
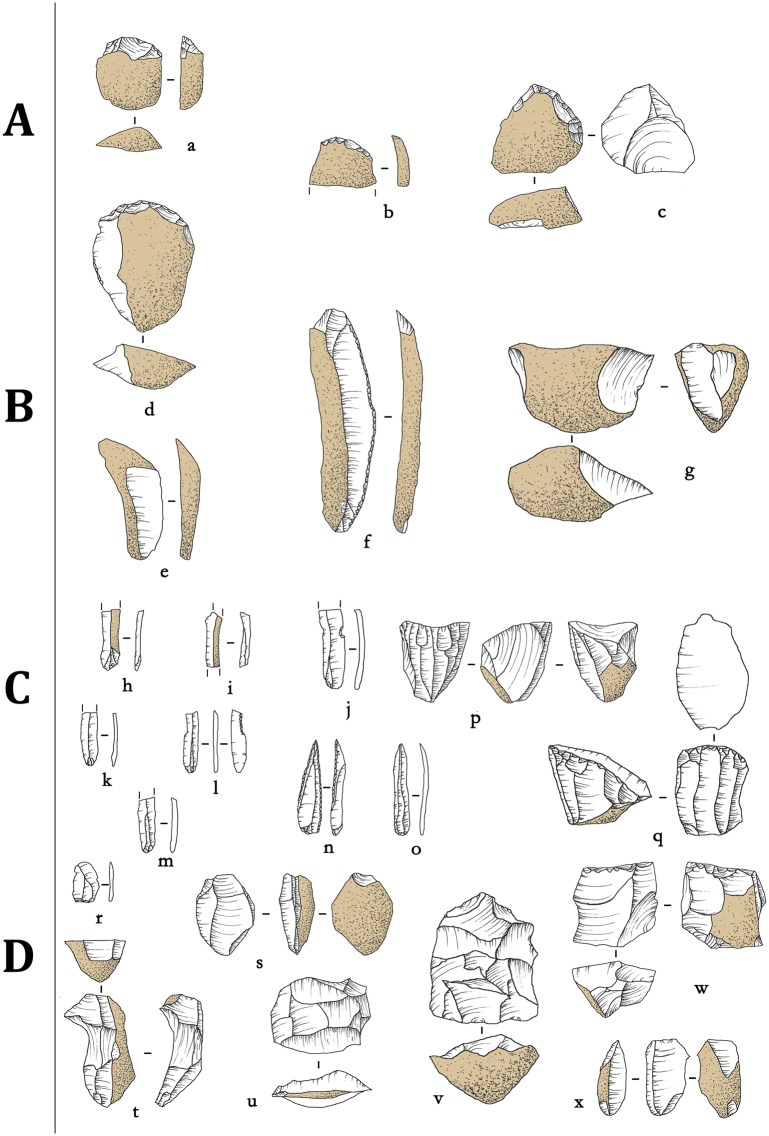
Shizitan 29, layer 7 Top: Reduction sequence. Lithic drawings showing reduction stages for River flint lithic industry from layer 7 Top. (a-c) Flakes from Stage A; (d-f) Flakes and (g) cores from Stage B; (h-o) Blades and (p-q) cores from Stage C; (r-u) Flakes and (v-x) cores from Stage D.

**Fig 6:**
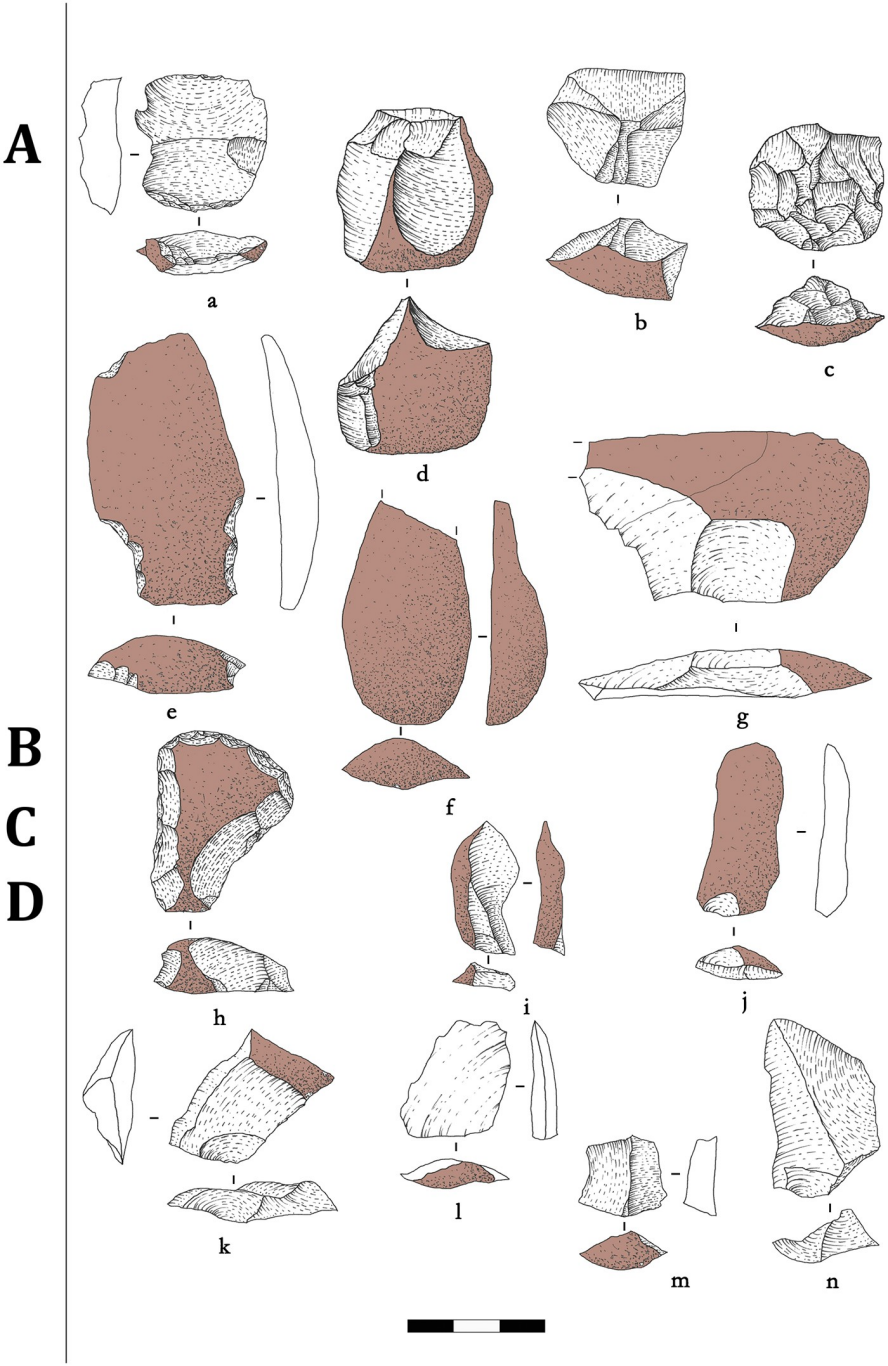
Shizitan 29, layer 7 Top: Reduction sequence. Lithic drawings showing reduction stages for Quartzite lithic industry from layer 7 Top. (a-c) Cores showing (a) evidence of anvil percussion; (e-n) Flakes showing a single reduction stage until the abandonment of the cores.

Layer 7 Top marks a clear technical change at Shizitan 29 with the sudden appearance of a true blade production. Black flint (more than 45%) (Fig 4) and River flint (about 40%) (Fig 5) are the most knapped raw materials, while quartzite (Fig 6) and other raw materials become less prevalent (about 14%). Notable is the increased presence of River flint when compared to the previous assemblage. Another significant change is that in this assemblage, cores are very abundant and are in association with a large number of blades. Also, other than raw material, there are no noticeable differences observed between cores or blades made from Black flint or River flint, so here we describe the cores and the blades each as one group.

In the following discussion, we will distinguish between “elongated blanks” and “blades” from a technical perspective: “elongated blanks” show irregular (i.e., not parallel) edges/nervures while “blades” show parallel or sub-parallel, morphologically standardized edges/nervures.

**Fig 7:**
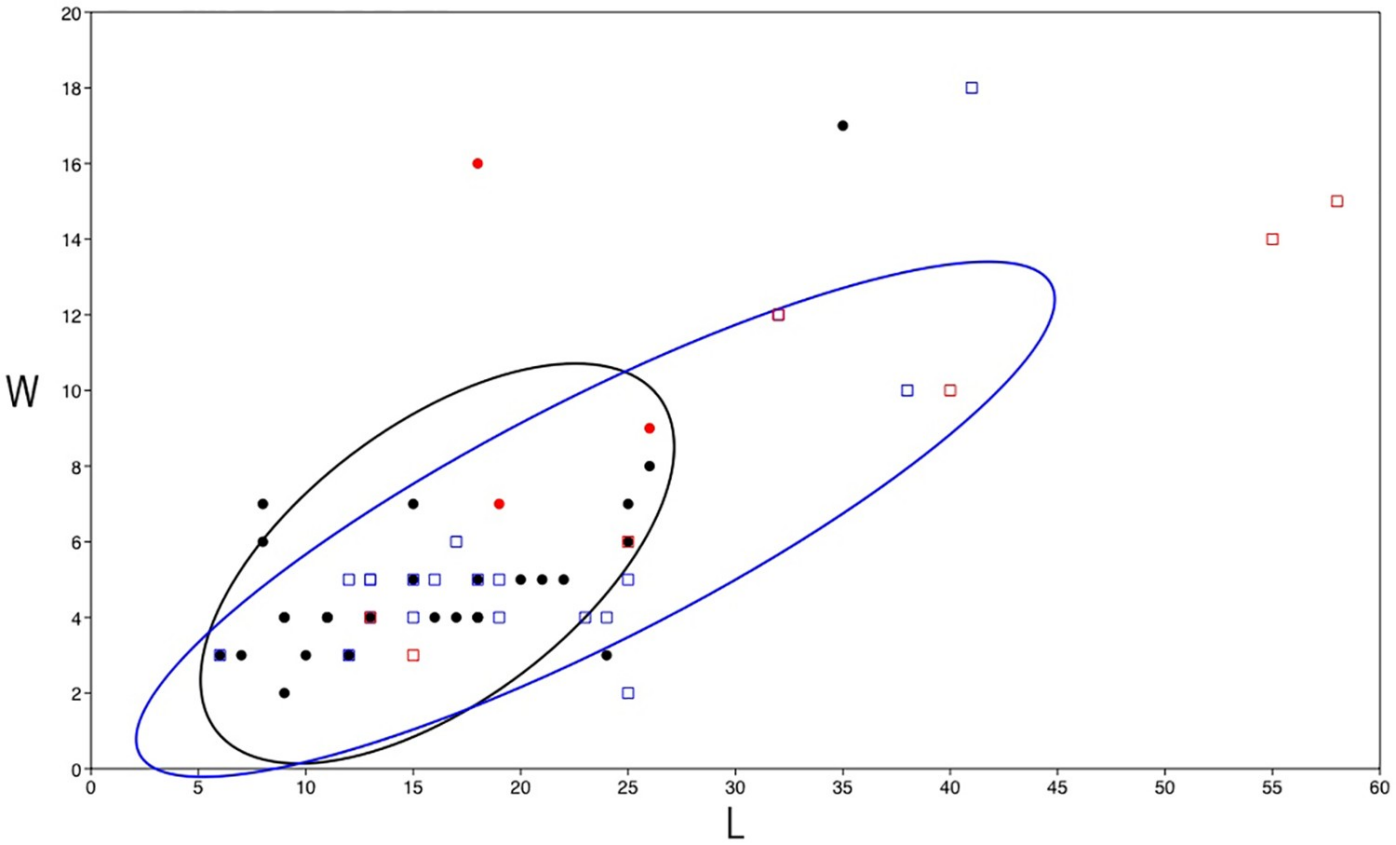
Shizitan 29, layer 7 Top: Dimensional analysis. (L) Length and (W) Width ratio of unbroken blades from layer 7 Top with concentration ellipses of 70%. (Black dot) Black flint; (Red dot) retouched Black flint; (Blue square) River flint; (Red square) retouched River flint.

**Fig 8:**
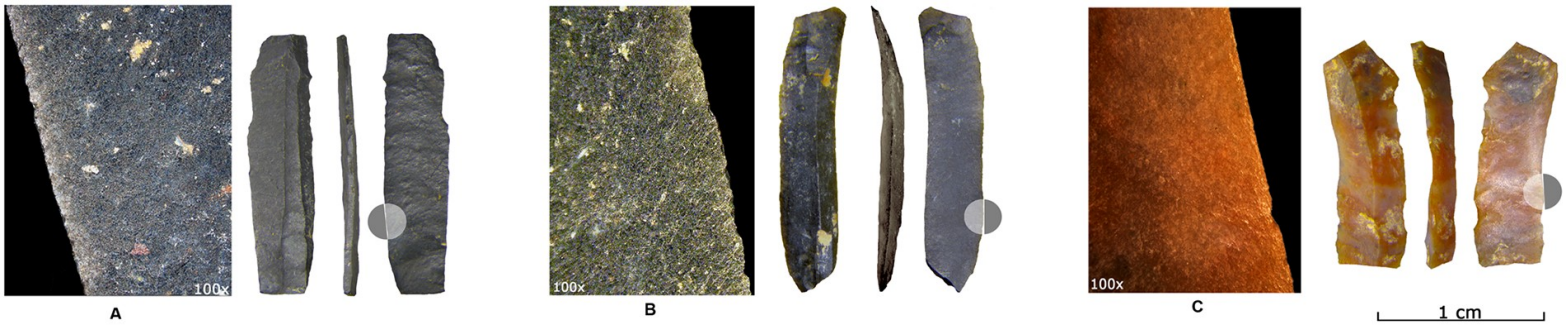
Shizitan 29, layer 7 Top: Functional analysis. Blades from layer 7 Top showing use wear traces (cutting actions on different materials).

About 50% of the cores present evidence for the production of flakes; around 30% show clear blade production; and less than 20% show elongated flake production. These core-derived data stand in contrast to the abundant presence of blades in this assemblage (especially considering we only counted blades from the 150 m^2^ sampling zone but counted cores from the entire layer). Significantly, blades are morphologically standardized regardless of raw material. If we consider the length/width ratio (Fig 7), the unbroken blades (70 blades) are no longer than 25 mm and range from two to eight mm in width. Some outlier blades, characterized by their size exceeding the standard dimensions of the assemblage, are present but are represented only by retouched and/or half-cortical blades (Fig 5f). As their dimensions are larger than other blades and too large to have been produced by the cores present in the layer, they seem to be imported into the site. Most blades show straight profiles (67% from Black flint; 46% from River flint) but twisted (13% Black flint; 11% from River flint) and convex (13% for Black flint and 19% for River flint) profiles are also observed. From a technical perspective, twisted and convex blades should be considered as by-products useful in maintaining the working surface of the core in order to allow the production of blades with a straight profile. Only 36 blades (16.5%) show retouched edges (Table 4) (Figs 4b, 5j and 5n), with 10 of them showing marginal retouch (Figs 4a and 5l). Preliminary functional observations (Fig 8) on a sample of blades reveal macro and micro use wear related to different materials but mainly for cutting activities. Thus, we suggest that the aim of blade production was for such purposes (rather than as projectiles, scrapers, or drills [36]), although complete study is still needed. In contrast to blades, almost all flakes have been retouched, regardless of their reduction stage attribution. Black flint retouched flakes are much more common in the final two stages (C and D), while most retouched flakes on River flint belong to the first two stages of the reduction sequence. Compared to this, 77 retouched flakes represent 44% of the collected flakes; ten elongated flakes have been retouched as endscrapers (Figs 5a–5d and 6h), while scrapers and marginal retouch characterize most of the other retouched flakes (Fig 6h).

**Table 4:** Shizitan 29, layer 7 (top): The reduction sequence.

**Retouched blade**	**Stage A**	**Stage B**	**Stage C**	**Stage D**	
**BF**	0	3	13	0	**16**
**RF**	0	4	16	0	**20**
**Q**	0	0	0	0	**0**
	**0**	**7**	**29**	**0**	**36**
**Retouched flakes**	**Stage A**	**Stage B**	**Stage C**	**Stage D**	
**BF**	4	14	14	12	**44**
**RF**	8	10	5	4	**27**
**Q**	8	5	8	5	**26**
	**20**	**29**	**27**	**21**	**97**

Frequencies of retouched blanks and their reduction stage attribution, from Shizitan 29, layer 7 Top according to raw materials: (BF) Black flint; (RF) River flint; (Q) quartz/quartzite; (O) other materials.

### Reduction sequence: General aspects (Layer 7 Top)

All cores but one have been attributed to a reduction stage; 222 out of 230 blades and 94 out of 175 flakes also have been attributed (Table 5). All reduction stages are observed, but there are differences in distributions by raw material. Cores made from River flint are more or less equally distributed among the stages. For Black flint, however, Stage A is missing among cores. Black flint cores are particularly abundant in Stage C, and most should be seen as completely exploited blade cores. A very scanty presence of quartzite pebbles—generally exploited for flake production—is observed.

**Table 5:** Shizitan 29, layer 7 (top): The reduction sequence.

	Stage A			Stage B			Stage C			Stage D			
	C	B	F	C	B	F	C	B	F	C	B	F	
**BF**	0	0	4	8	5	14	21	98	9	4	0	12	**175**
**RF**	4	0	8	7	18	10	7	93	5	8	0	4	**164**
**Q**	0	0	8	1	0	5	5	0	8	1	1	5	**34**
**O**	0	0	1	0	1	0	0	6	0	0	0	1	**9**
	**4**	**0**	**21**	**16**	**24**	**29**	**33**	**197**	**22**	**13**	**1**	**22**	**382**

Reduction stages of attributable (C) cores, (B) blades, and (F) flakes from Shizitan 29, layer 7 Top according to raw materials: (BF) Black flint; (RF) River flint; (Q) quartz/quartzite; (O) other materials.

Generally speaking, the results show that River and Black flints are mainly knapped by direct percussion and pressure technique in order to produce blades. In contrast to this, quartzite pebbles are only knapped to produce flakes. Anvil percussion is not well represented and has no clear relation to a specific raw material. We also suggest that the presence of flake and blade production together needs to be further considered and investigated. For example, Black flint shows a mixture of flake and blade production, but this could possibly be explained by a need to produce flakes in order to pre-form the blade cores. The use of River flint cores also needs further consideration, as some cores made from River and the “other” flints do not seem strictly related to blade production, as they are present in all reduction stages, particularly Stage D.

**Fig 9:**
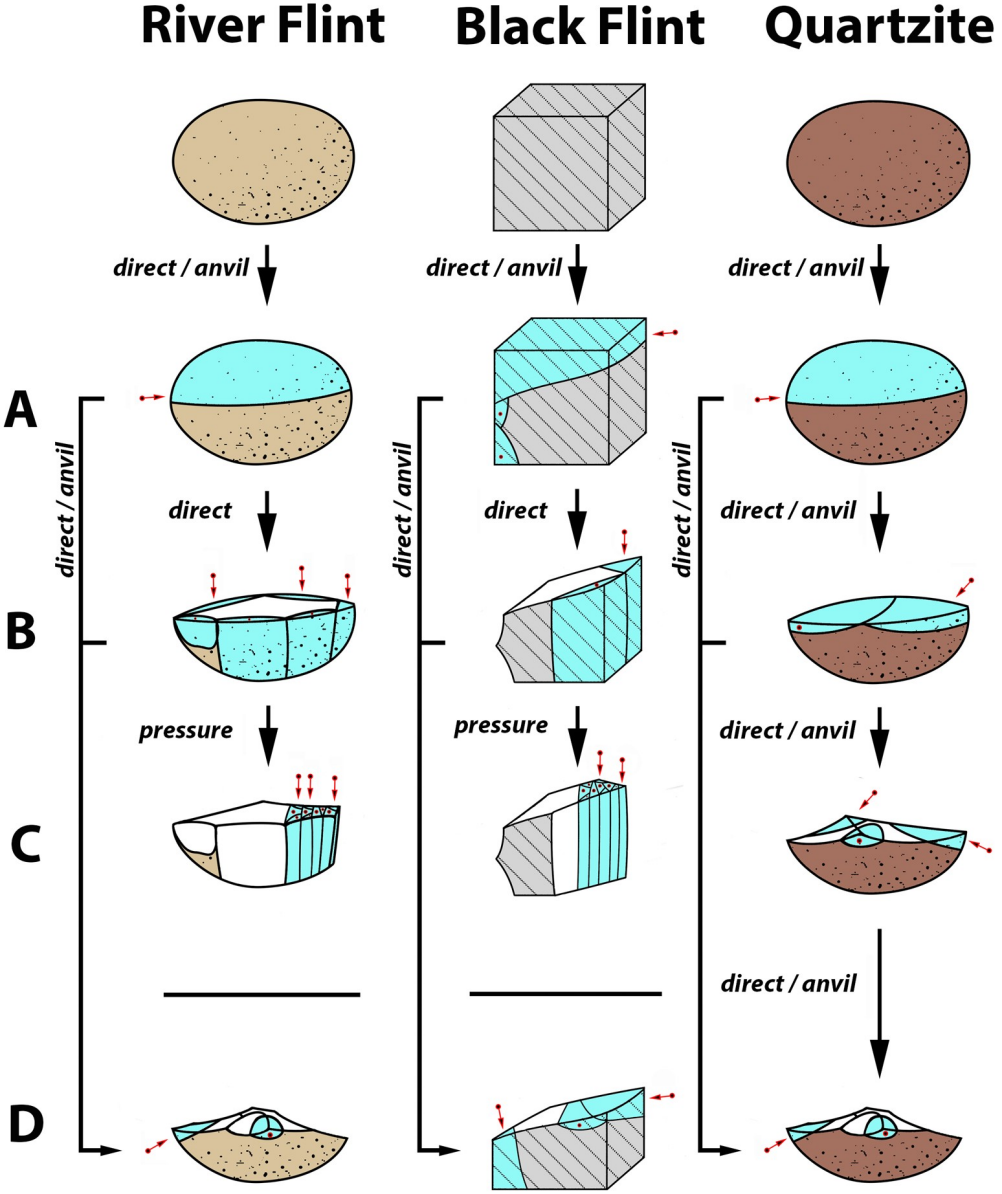
Shizitan 29: The reduction sequence. A reconstruction of the reduction sequence recognized in layer 7 Top divided by raw materials and reduction.

To better understand the significance of blade production, its potential origins, and its evolution following its initial appearance in Shizitan 29, deeper insight can come from the reconstruction of reduction sequences. Following the schematic reduction model (Fig 9), in Table 6 we present a detailed reduction sequence for the Shizitan 29 Black flint, River flint, and quartzite. Future studies at other sites following this approach can then be compared to gain further evolutionary insights.

**Table 6:** Shizitan 29: The reduction sequence.

	Black flint	River flint	Quartzite
**Stage A**	This stage is not observed. Probably Black flint is imported into the site as pre-formed cores or as large, thick flakes to be used as cores.	Pebbles may be splitted by means of anvil percussion or, more probably, by detaching a large thick flake by means of direct percussion. The flake and the residual pebble may both be used as a blade core.	Pebbles may be splitted by means of anvil percussion or, more probably, by detaching a large thick flake by means of direct percussion. The flake and the residual pebble may both be used as a core.
**Stage B**	The core is used, or the ventral surface of a flake is used as the striking platform, each of which has very little—if any—preparation throughout the knapping sequence. By direct percussion, unidirectional elongated flakes are produced so as to shape the core in order to obtain a pre-formed blade core. From this stage, the core may also be exploited in order to produce only flakes or elongated flakes; in this case the reduction sequence may continue until Stage D.		Flake production by direct percussion (or even by anvil percussion) to exploit the core until its abandonment.
	The natural morphology of Black flint (squared blocks or irregular nodules) requires a more developed preparation. Preparation includes flakes or elongated flakes that are detached from any side of the core. This provides an angulated side to the core that will be exploited for the beginning of the blade production.	The natural morphology of River flint (rounded pebbles) does not require a more developed preparation	
**Stage C**	The production of blades starts. No maintenance of the core is needed. The core is abandoned at this stage only when completely exploited or because of knapping mistakes. The blades are usually detached from one side of the core: this mainly allows the production of blades with a straight profile.		
	Maintenance of the core is more complex when Black flint is knapped. Following the natural morphology of the core, several technical products must be occasionally detached in order to continue the blade production. These include blades with a naturally backed edge and flakes or elongated flakes from different areas of the core: their removal keeps the working surface as large as possible, provides crests, and so on.	The natural morphology of River flint (rounded pebbles) does not require a complex maintenance of the core	
**Stage D**	When blade production is over, but the core is not completely exhausted, it can be exploited by direct percussion (less probably by means of anvil percussion) to produce more flakes. Similarly, flake cores can reach this stage directly from Stage B.		

Description of the reduction sequence stages for the Black flint, River flint, and quartzite at Shizitan 29

## Discussion

Shizitan 29 provides the earliest, well-dated evidence in East Asia for the appearance of the tiny, morphologically standardized blades commonly referred to in the literature as “microblades”. Our techno-functional analysis results indicate that these blades were probably produced by pressure technique following a heat-treatment of the cores (analyses at this regard are still ongoing). Significantly, the site does not show evidence for the local emergence and development of the new operational sequence required for the production of these blanks: instead, our approach leads us to inter that the operational sequence first appears in an already developed form. Thus, this approach allows us to see this evidence as an introduced or intrusive technology.

Where, how, and why the so-called “microblade” technology emerged has remained poorly understood, as have the relationships of microblade production between the different cultural regions where it spreads by the Terminal Pleistocene and the reasons for the widespread distribution of this specialized technology. In China, few Upper Paleolithic assemblages have been formally published, and in our opinion, the lithic evidence is typically described in techno-typological terms, the basis for which is exclusively morphological [37–39]. As a result, little information is provided about technological features of the analyzed assemblages. This impacts our ability to determine the origins of this technology and the pathways of its evolution. Furthermore, the technological significance of microblade production (particularly versus other blade productions) remains under-discussed and under theorized.

The traditional typological approach [40] continues to permeate the way technological analyses are carried out, with new “technological” typologies merely replacing the old “morphological” ones without the necessary consideration of technical objectives. A clear example is given by the lack of a standardized usage for the term “microblade”: a shared technical and/or dimensional definition of the term is still missing as studies define microblades and cores only on the basis of metrical and morphological features established case by case, or they follow differing definitions [41–42] which can result in the arbitrary classification of borderline cases and obfuscate similarities in technological aspects [12,43–47].

Thus, in the Chinese literature, with the traditional typological approach, “blades”—also referred to as “typical blades” or “big blades” [46–48]—are identified by measurement standards, especially by length and width: generally speaking, a blade is defined as being at least twice as long as it is wide (and with the width usually having to be more than 12 mm) [46]. Some authors also focus attention on thickness [49–50]. Recently, following the Western literature, other standards, including edges, ridges, knapping technology, and so on, are sometimes taken into account, but size still continues to be the dominant character to identify blades. As far as the reduction sequence is concerned, only two blade reduction methods have been identified in northern China, and both on the basis of core morphology: the Levallois and the prismatic methods [51]. Outside of China, “bladelets” are generally considered as something dimensionally in-between the production of blades and microblades, and the bladelets are considered an evolved blade technology that occurs with the global microlithization of stone tools. However, we only see this consideration of bladelet assemblages as an “in-between” technology in foreign references [50], but none in Chinese references. “Microblades” are sometimes also viewed as a specialization of blade or bladelet production in certain regions of China [50], especially in the use of indirect/pressure flaking techniques. Even though some technological descriptions have been applied to microblade assemblages in the Chinese literature (using such terms as “elongated blanks” or “reduction sequence” [24]), the technical, quantitative, or qualitative data needed to clearly describe the adaptive role played by the suggested core reduction models are lacking [39].

Another problem of the traditional typological approach across East Asia is that analyses of the suggested microblade assemblages solely rely upon characterization of the cores’ final shape. As a result, in this traditional approach, the only items analytically relevant are those that we would identify as being in Stage C of the reduction sequence, as these are the cores that can be classified according to their final shapes (i.e., their shape at abandonment and deposition into the archaeological record only after a full number of blades have been removed, as well as without having been reshaped by Stage D production). These cores have been given such type names as “wedge-shaped”, “boat-shaped”, “funnel”, “cylindrical”, “conical”, or even “semi-cylindrical” and “semi-conical” cores [52–56]. Sometimes, further description or sub-classifications are added that allude to assumed technological productions, but these cores are still analyzed and named according to final shape, such as “wide wedge-shaped microcores” [49]. The behavioral and evolutionary reconstructions and understandings that can be drawn from the type clusters created by this exercise are limited. More importantly, this methodology makes little or no consideration of potential differences in the reduction sequence or other technological aspects of production that can have great behavioral significance.

Furthermore, inadequate attention has been given to raw material procurement strategies, to models of curation vs. expediency, and to techno-functional differences in the production of tools that could relate to raw material variability and/or to the exploitation of different raw material sources. In fact, interpretation of assemblages is limited to generalized statements concerning assumed adaptive benefits for functions only inferred through morphology. These assumed functional advantages then serve as explanation for both the origin of a still undefined microblade technology and its rapid spread [36]. Our approach to the Shizitan 29 lithic assemblages overcomes the problems of simply making morphological distinctions between blades and microblades without techno-functional considerations, as well as the problems of analytically separating fully-shaped microcores from cores still in their preliminary preparations, as we place the entire production sequence and its technical objectives to the fore: since these are more directly related to the prehistoric behaviors and concerns of the producer of the lithics than typological classification by the archaeologist is, this approach thus provides better support for our understanding of prehistoric behavior. In this analysis, in fact, the reason we do not first distinguish a “microblade” class from a “blade” class by an arbitrary size cut-off is that our interest is *behavioral*, and for this, it is more important first to place these tools inside their production sequences: this becomes the means to investigate and compare the mental processes and needs of the past human groups and then determine if they were distinguishing between these two potential classes of tools. In other words, in re-thinking the evolution of microblade technology, our emphasis on production sequences allows us to sketch the various ways prehistoric groups satisfied specific needs, and this is done through understanding technological objectives.

By this point of view, in Layer 8 and Layer 7 Base, a clear “core-and-flake” lithic industry with no evidence of blade technology is attested. In Layer 8 the production of flakes is strictly associated with anvil and direct percussion with the objective of producing flakes with cutting edges. Here, the abundance of Black flint suggests mobility ranging east-west along the Qingshui River valley, between the site and known raw material sources: this range links the two major drainages of Shanxi, the Fen and the Yellow Rivers. The reason for the presence of quartz/quartzite pebbles and the paucity of River flint pebbles, when both can be collected from the Yellow River’s banks, remains to be explained. One possibility is a need for quartz/quartzite as a raw material with qualities significantly different from the Black flint. A follow-up hypothesis then links the combination of imported flint and local quartz/quartzite to specialized activities that must be carried out at the site. Consequently, it is possible to suggest that Layer 8 could represent the locality being used as a temporary, somehow functionally-specialized campsite. From a technological perspective, the “core-and-flake” assemblage found in Layer 7 Base is similar to that found in Layer 8. However, there are significant behavioral differences indicated by the greater abundance of flakes, the paucity of cores, and the presence of local, River flint: these may be seen as evidence for a different use of the site.

From Layer 7 Top to Layer 1 [1,21], the lithic sequence is characterized by the presence of blade production by means of pressure technique. Layer 7 Top itself shows a mixture of both lithic productions: a “core-and-flake”-like assemblage along with a clear blade technology that appears suddenly and fully developed. Here, while flakes are still present, in contrast to Layers 8 and 7 Base, the assemblage-level dataset allows us to interpret the flake production as a by-product of blade production. Moreover, blade features (such as their profile, cortex distribution, dimensions, and types of butt) suggest that the earliest blades in Shizitan 29 may have been produced by pressure technique [57–58] and heat treatment possibly may have been adopted (we are currently investigating this). Also, Black flint and good quality River flint are knapped following similar procedures. This blade production appears suddenly and fully developed here: the data indicate that blade production is intrusive in the site and represents a significant change without any evidence of local transition from what appears before it. It is still difficult to say if the intrusion is caused by the presence of new people at the site or by the introduction of new technical know-how that completely replaces previous lithic productions.

Finally, the presence of larger blades (but still within the size range of what morphologically could be termed “microblades” in traditional approaches) (see an example in Fig 5f) made from River flint in Layer 7 Top is very interesting, as the local River flint consists only of small pebbles that could not produce blades of this size. An intriguing explanation for these “giant” blades could be that they were produced elsewhere and introduced into the site as finished products. Where they were made would likely have been further northward along the Yellow River, where larger River flint pebbles may be found. This hypothesis may also be supported by the earlier appearance of blade technology (with forms larger than those commonly called “microblades”) in areas of China further north, such as at Shuidonggou [37], as well as even earlier and further north in the Altai [10, 47, 59].

In conclusion, rather than applying traditional morphological typology to the Shizitan 29 lithic assemblage, this study serves to demonstrate the benefits of a technological analysis focusing on reduction sequence and raw material provenience. Following this study, we suggest that the application of this methodology and approach with its theoretical underpinnings to other Upper Paleolithic sites of the Late and Terminal Pleistocene in North China and other areas in northeastern Asia, particularly to well-dated assemblages, would have the potential to contribute greatly to our understanding of the origins, evolution, and spread of blade technology and particularly to the adaptive relationships between the so-called microblade productions with each other and with earlier blade production and other East Asian lithic technologies.
